# Progress in Understanding Algal Bloom-Mediated Fish Kills: The Role of Superoxide Radicals, Phycotoxins and Fatty Acids

**DOI:** 10.1371/journal.pone.0133549

**Published:** 2015-07-21

**Authors:** Juan José Dorantes-Aranda, Andreas Seger, Jorge I. Mardones, Peter D. Nichols, Gustaaf M. Hallegraeff

**Affiliations:** 1 Institute for Marine and Antarctic Studies, University of Tasmania, Hobart, Tasmania, Australia; 2 CSIRO Oceans and Atmosphere Flagship, Hobart, Tasmania, Australia; Stony Brook University, UNITED STATES

## Abstract

Quantification of the role of reactive oxygen species, phycotoxins and fatty acids in fish toxicity by harmful marine microalgae remains inconclusive. An *in vitro* fish gill (from rainbow trout *Oncorhynchus mykiss*) assay was used to simultaneously assess the effect in superoxide dismutase, catalase and lactate dehydrogenase enzymatic activities caused by seven species of ichthyotoxic microalgae (*Chattonella marina*, *Fibrocapsa japonica*, *Heterosigma akashiwo*, *Karenia mikimotoi*, *Alexandrium catenella*, *Karlodinium veneficum*, *Prymnesium parvum*). Quantification of superoxide production by these algae was also performed. The effect of purified phycotoxins and crude extracts was compared, and the effect of fatty acids is discussed. The raphidophyte *Chattonella *was the most ichthyotoxic (gill cell viability down to 35%) and also the major producer of superoxide radicals (14 pmol cell^-1^ hr^-1^) especially after cell lysis. The raphidophyte *Heterosigma *and dinoflagellate *Alexandrium *were the least toxic and had low superoxide production, except when *A*. *catenella* was lysed (5.6 pmol cell^-1^ hr^-1^). Catalase showed no changes in activity in all the treatments. Superoxide dismutase (SOD) and lactate dehydrogenase exhibited significant activity increases of ≤23% and 51.2% TCC (total cellular content), respectively, after exposure to *C*. *marina*, but SOD showed insignificant changes with remaining algal species. A strong relationship between gill cell viability and superoxide production or superoxide dismutase was not observed. Purified brevetoxins PbTx-2 and -3 (from *Karenia brevis*, LC_50_ of 22.1 versus 35.2 μg mL^-1^) and karlotoxin KmTx-2 (from *Karlodinium*; LC_50_ = 380 ng mL^-1^) could almost entirely account for the fish killing activity by those two dinoflagellates. However, the paralytic shellfish toxins (PST) GTX1&4, C1&C2, and STX did not account for *Alexandrium *ichthyotoxicity. Only aqueous extracts of *Alexandrium *were cytotoxic (≤65% decrease of viability), whereas crude methanol and acetone extracts of *Chattonella*, *Fibrocapsa*, *Heterosigma*, *Karlodinium* and *Prymnesium *decreased cell viability down to 0%. These and our previous findings involving the role of fatty acids confirm that superoxide radicals are only partially involved in ichthyotoxicity and point to a highly variable contribution by other compounds such as lipid peroxidation products (e.g. aldehydes).

## Introduction

The multi-million dollar economic impacts from harmful microalgae on fish farming have been well documented and far exceed the comparable impacts on shellfish industries, where the main impact is direct loss of the product due to mass mortalities [1–5]. For instance, the economic losses in Japan between 1972 and 2012 were 34.2 billion JPY (~US$352 million) and 6.9 billion JPY (~US$71 million) for the finfish and shellfish industries, respectively [6]. The species that have created highest impacts on fish farms worldwide are the unarmoured *Cochlodinium polykrikoides*, *Karenia mikimotoi* (dinoflagellates), *Chattonella marina*, *C*. *antiqua* and *Heterosigma akashiwo* (raphidophytes), *Prymnesium parvum* (haptophyte); more rarely the armoured dinoflagellates *Karlodinium veneficum*, *Alexandrium catenella* and *A*. *tamarense* have also caused adverse effects [7]. Despite the impacts of these events, it remains to be clarified how these microalgae that do not produce chemically characterized toxins (different from neurotoxic, diarrhetic, amnesic and paralytic shellfish poisoning causing metabolites) are killing finfish.

Ichthyotoxicity has been attributed variously to production of free fatty acids [8–10] and reactive oxygen species or ROS (the superoxide radical O_2_^-^ in particular) [11,12] and occasionally to chemically defined phycotoxins such as brevetoxins or karlotoxins [13–15]. However, not all ichthyotoxic microalgae produce these compounds in amounts that can account for their impacts on fish.

Reactive oxygen species are the result of electron transport, as occurs in metabolic processes within the cell. If molecular oxygen (O_2_) accepts a single electron, the product is the superoxide radical; when O_2_^-^ is reduced by a second electron, hydrogen peroxide (H_2_O_2_) is produced, and if reduction of H_2_O_2_ by a third electron occurs this can then lead to generation of hydroxyl radicals (OH•) [16]. Reactive oxygen species are produced during respiration and photosynthesis, and can be significantly reduced using photosynthesis blockers, which suggests that fish mortality may be more prominent during day light hours [17].

Sensitive assays have been developed to quantify superoxide and hydroxyl radicals in aqueous solution. Both radicals have been detected in seawater; however, due to the high reactivity of hydroxyl, and thus its short lifetime (~μs) [18,19], only the superoxide anion can be accurately measured in cultures of microalgae [11,20–22]. Superoxide lifetime in seawater has been measured in the range of 10–300 s [19]. The improved MCLA (2-methyl-6-(4-methoxyphenyl)-3,7-dihydroimidazo[1,2-a]pyrazin-3(7H)-one) assay adapted by Godrant et al. [22] offers the advantage of performing superoxide tests on microplates, without having to use large volumes of algal cultures and hence enables better replication and also simultaneous measurements. This assay is based on the chemilumninescence of MCLA generated when it reacts with the superoxide radicals in the medium, as being produced by the algae. The signal is measured by a luminescence detector using a microplate reader.

Screening for generation of superoxide by a wide range of microalgae, has conclusively shown that raphidophytes of the genus *Chattonella* are the greatest O_2_^-^ producers, generating up to 18 times more superoxide than other ichthyotoxic species, including raphidophytes and dinoflagellates [21]. There exists controversy in the ichthyotoxic mechanism of the dinoflagellate *Cochlodinium polykrikoides*, since early studies suggested the role of ROS but others found only trace levels, concluding that *C*. *marina* and *C*. *polykrikoides* have different toxic mechanisms [23–25]. Ichthyotoxic unarmoured microalgae are very fragile and susceptible to cell rupture; when this occurs, a cocktail of reactive compounds are released into the water. These compounds affect the fish mainly via gill damage during respiration, and this damage can be accelerated with hyperventilation under continuing stress conditions [12]. With some species, such as the armoured dinoflagellate *Karlodinium*, cell lysis has been conclusively demonstrated to be critical for ichthyotoxicity [26], and similarly for the naked flagellate *Chattonella* more fragile strains are more potent fish killers [27].

Living organisms possess enzymatic antioxidant defenses to ROS, such as catalase, glutathione peroxidase, superoxide dismutase, as well as the non-enzymatic vitamin C and E, and β–carotene. However, when ROS production exceeds these defenses, organisms can undergo oxidative stress through damage to proteins, DNA and lipids, creating physiological changes that may lead to death. Thus the activity of ROS defense enzymes may serve as a biomarker for ROS exposure [28]. Similarly, in pioneering fish challenge experiments by Yang et al. [29], the ROS biomarkers catalase and superoxide dismutase appeared to protect rainbow trout against *Heterosigma akashiwo* (reported as *A*. *carterae)*.

In our previous experimental studies we presented a sensitive *in vitro* assay to test toxicity of harmful phytoplankton using a gill cell line from rainbow trout *Oncorhynchus mykiss* as a model [27,30]. The premise of this approach is that fish gill damage is the first line of attack in harmful microalgae killing fish [31]. In the present study, we challenged the gill cells against different harmful microalgae and measure the activity of antioxidant enzymes in the gill cells, as well as superoxide production by the microalgae. The effect of purified phycotoxins and crude algal extracts was also assessed in gill cells to resolve the role of superoxide radicals, phycotoxins or other algal compounds in ichthyotoxicity.

## Materials and Methods

### Fish gill cell line culturing

The gill cell line RTgill-W1, derived from rainbow trout *Oncorhynchus mykiss* [32], was obtained from the American Type Culture Collection (CRL-2523). This cell line was routinely cultured on 25-cm^2^ culture treated flasks (690170, Greiner Bio-One) with Leibovitz’s medium (L1518, Sigma) supplemented with 10% fetal bovine serum (v/v) (12003C, Sigma) and an antibiotic-antimycotic solution (A5955, Sigma). Cells were incubated at 20°C in the dark. This epithelial cell line was detached with 0.25% trypsin-0.02% EDTA solution (59428C, Sigma) for subculturing and seeding purposes [30].

### Microalgal species

Seven species of ichthyotoxic microalgae, and one nontoxic species, were obtained from the Harmful Algal Culture Collection of the Institute for Marine and Antarctic Studies (University of Tasmania). Algae were grown in GSe medium [33] using seawater at 35 salinity, and kept at 20°C (except for strain ACCH05 that was grown at 17°C) under a 12:12-hr light:dark cycle at 150 μmol photons m^-2^ s^-1^ of light intensity (cool white fluorescent lamps). Details of all species are shown in Table 1.

**Table 1:** Species of marine phytoplankton used for exposure experiments to test their toxicity on fish cells RTgill-W1, and production of superoxide radicals. The nontoxic species *Tetraselmis suecica* was used as a negative control. Two strains of the raphidophyte *Chattonella marina* were used.

Class/Species	Strain code	Origin	Isolator (year)	Original Source
Chlorophyceae				
* Tetraselmis suecica*	TSCS187	Brest, France	A. Dodson	CSIRO Microalgal supply (original collection CCMP), Aus.
Dinophyceae				
* Alexandrium catenella*	ACCH05	Isla Ceres, Aysen, Chile.	A. Zuñiga (2009)	Centro de Investigación y Desarrollo de Recursos de Ambientes Costeros, I-Mar, Chile (original strain code: CERES8).
* Karenia mikimotoi*	KMWL01	West Lakes, S.A., Aus.	M. de Salas (2006)	University of Tasmania, Aus.
* Karlodinium veneficum*	KVSR01	Swan River, W.A., Aus.	M. de Salas (2001)	University of Tasmania, Aus.
Prymnesiophyceae				
* Prymnesium parvum*	PPDW02	Darwin, N.T., Aus.	G. Hallegraeff (2009)	University of Tasmania, Aus.
Raphidophyceae				
* Chattonella marina*	CMPL01	Port Lincoln, S.A., Aus.	J.A. Marshall (1996)	University of Tasmania, Aus.
	N-118	Seto Inland Sea, Jap.	S. Yoshimatsu (1983)	National Institute of Environmental Studies, Jap.
* Fibrocapsa japonica*	FJCS332	Tsuda Bay Kagawa, Jap.	K. Yuki (1978)	CSIRO Microalgal supply (original collection CCMP), Aus.
* Heterosigma akashiwo*	HAGB01	Georges Bay, Tas., Aus.	M. de Salas	University of Tasmania, Aus.

Algal cultures were used at three concentrations (designated high, medium, and low), which were prepared by taking dense cultures from the exponential growth phase either unconcentrated or diluted by one and two thirds. One batch of the cultures was used without any treatment prior to exposure (whole cells), while another batch was sonicated to rupture algal cells (lysed cells). All cultures were used for the exposure of gill cells to living or ruptured algae, and for enzymatic analysis in gill cells.

### Preparation of crude algal extracts

Algae were grown in 400 mL flasks under the same conditions as described above. Aliquots of 30 mL of cultures in the exponential growth phase were transferred into five tubes and centrifuged at 805 ×g for 10 min. The supernatant was discarded and 2 mL of solvent: milliQ water, acetic acid 30 mM, hydrochloric acid 1 mM, methanol 99%, or acetone 80%, was added to the tubes (one solvent per tube). These solvents possess different polarity and thus are able to extract different kind of compounds (i.e. water and acids can extract more hydrophylic compounds due to their high polarity, and alcohols can extract some polar but also non polar compounds at some extent, such as fatty acids). Pellets were resuspended in each solvent (milliQ water, acetic acid, hydrochloric acid, methanol, or acetone) and sonicated for 10 min in an ice bath. Samples were centrifuged and the supernatant stored at -80°C.

### Exposure experiments

#### Exposure of gill cells to living algae

Confluent gill cells were detached from flasks with trypsin, counted and adjusted to 9×10^4^ cells mL^-1^. From this, 80 μL of gill cells in suspension was seeded in quadruplicate in 96-well microplate with inserts (CLS3381, Sigma) 48 hrs prior to the experiments. The lower compartment was filled with 150 μL of L-15/ex medium [34] at a salinity of 30, and a volume of 80 μL of algal culture was added to the inserts and incubated for 2 hrs at 20°C and 150 μmol photons m^-2^ s^-1^.

#### Exposure of gill cells to crude algal extracts and purified phycotoxins

Confluent gill cells were detached, counted and adjusted to 2×10^5^ cells mL^-1^; 100 μL of gill cells in suspension was seeded in quadruplicate in 96-well conventional microplates (655180, Greiner Bio-One). Algal extracts were dissolved in L-15/ex medium at concentrations of 0.2, 0.4, 0.7, 0.9, 1.1 and 2.2% (v/v) (final concentrations of solvents were: acetic acid 0.3 mM, HCl 11 μM, MeOH 1%, and acetone 0.9%). Purified brevetoxins, PbTx-2 and PbTx-3 (MARBIONC, USA), and karlotoxin KmTx-2 (provided by Allen R. Place) were dissolved in MeOH 99% and mixed with L-15/ex to final concentrations of 0.1, 1, 10, 20, 40 μg mL^-1^ (brevetoxin); 0.021, 0.21, 2.1, 21, 210 ng mL^-1^ (equivalent karlotoxin production by *K*. *veneficum*, tested at pH 7.5 and 9.0 with a control group for each pH), another batch of gill cells was exposed to karlotoxin at 0.1, 1, 10, 100, 1000 ng mL^-1^. PST toxins, gonyautoxins 1 and 4 (GTX1&4-c), N-sulfocarbamoyl-gonyautoxin 2 and 3 (C1&C2-b) and saxitoxin (STX-f) (National Research Council Canada), were tested at varying concentrations. Gill cells were rinsed with phosphate buffer saline (PBS), L-15/ex containing the algal extracts or toxins were added and incubated at 20°C and 150 μmol photons m^-2^ s^-1^. Exposure of gill cells to algal extracts, brevetoxins and PST toxins was for 2 hrs, and to karlotoxin for 2, 3, 4 and 5 hrs.

#### Viability assay in gill cells

Algal cultures, algal extracts and phycotoxins were discarded from the plates after the exposure, and gill cells rinsed with PBS. The indicator dye alamar blue (DAL1025, Invitrogen) in L-15/ex (5% v/v) was used to measure gill cell viability. The oxidized form of alamar blue (resazurin, blue in color) is a non-fluorescent substrate that is metabolised by live cells and reduced to a fluorescent pink compound (resorufin), thus reflecting cell viability [35,36]. Alamar blue was added to all wells with gill cells, and incubated for 2 hrs in the dark [37]. Fluorescence of metabolised alamar blue was measured with a microplate reader (FLUOstar OPTIMA, BMG Labtech, 413–3350), using excitation and emission filters of 540 and 590 nm, respectively. For a detailed protocol of these assays, see Dorantes-Aranda et al. [30].

#### Homogenate preparation of gill cells for enzymatic assays

Gill cells were seeded as previously described, except that concentrations were adjusted to 6×10^4^ cells mL^-1^, and 3 mL of this was transferred into duplicate 6-well plates with inserts (CLS3450, Sigma). Gill cells were exposed to algal cultures under the same conditions as above. The control treatment was gill cells exposed only to GSe medium. Once the 2-hr exposure was completed, algal cultures in the inserts were mixed and 1.5 mL aliquots transferred into Eppendorf tubes, and centrifuged at 370 ×g for 5 min. Supernatant was recovered and stored at -20°C until analysis of the enzyme lactate dehydrogenase (LDH) was performed to determine whether damage to cellular membrane occurred in association with changes in gill cell viability, as LDH activity is usually associated with cytotoxicity [38].

Immediately after discarding the rest of the algal cultures, gill cells were rinsed with PBS. One mL of PBS was added and cells detached with a cell scraper (CLS3010, Sigma). Cells suspended in PBS were transferred into Eppendorf tubes and centrifuged at 1000 ×g for 2 min. The supernatant was discarded and 500 μL of 50 mM potassium phosphate buffer (KPB) containing 1 mM ethylenediaminetetraacetid acid (EDTA) and 1 mM dithiothreitol (43816, Sigma) added. The pellet of gill cells was ground with a tissue grinder pestle (I1015-39, Astral Scientific) and mixed. Homogenate was centrifuged at 10 000 ×g for 10 min. The supernatant was transferred into Eppendorf tubes and stored at -20°C until analysis. This homogenate was used to determine protein concentration and activities of the enzymes catalase and superoxide dismutase.

#### Superoxide radical generation measurement in live and lysed algal cells

Production of superoxide radical by the algae used for the exposure experiments was measured according to Godrant et al. [22]. A volume of 270 μL of algal cultures was added to wells of a white opaque 96-well microplate (CLS3917, Sigma) containing 3 μL of 5 mM xanthine (X7375, Sigma). A blank containing xanthine, algal culture, and 5 kU mL^-1^ of superoxide dismutase (S7571, Sigma) was used for correction; a standard curve with xanthine, algal culture and different concentrations of xanthine oxidase (0.1, 0.7 and 1.5 U L^-1^) was also measured simultaneously. Finally, 5 μL of 125 μM MCLA (87787, Sigma) was injected to all wells, and luminescence signal was recorded for 20 min in a microplate reader (FLUOstar OPTIMA, BMG Labtech, 413–3350). The luminescence signal of each algal culture is calibrated against its respective standard curve with xanthine/xanthine oxidase, and the result divided by the algal concentration in the sample used, to then normalize and express the generation of superoxide as concentration of superoxide produced per cell per hr [22].

### Enzymatic assays in gill cells

#### Catalase, CAT

Catalase activity was measured according to Aebi [39], with a microplate adapted assay. KPB containing EDTA (final concentrations of 50 and 2.5 mM, respectively) at pH 7.4 was transferred into a 96-well microplate (655180, Greiner Bio-One), and 20 μL of the homogenate prepared from the gill cells was added, followed by 30 μL of 30 mM H_2_O_2_ (216763, Sigma). The mixture was mixed and the decrease of absorbance was measured at 240 nm for 5 min. Catalase activity was determined according to the following equation:

(ΔA240nmmin−1ε)(FinalvolumeSamplevolume)(1protein(mgmL−1))=mmolminm−1gprotein−1

ΔA is the slope of the linear regression obtained by plotting time (independent variable) versus absorbance (dependent variable).

ε is the molar extinction coefficient at 240 nm calculated for the microplate used for this assay.

#### Protein determination

Protein concentration in gill cell homogenate was determined with the standard colorimetric assay by Bradford [40]. The Protein Assay Kit II (500–0002) was purchased from Bio-Rad. Aliquots of 40 μL of the homogenate were added to wells of a 96-well microplate in triplicate, 160 μL of the dye reagent Coomassie Brilliant Blue was added to wells and incubated for 15 min at room temperature. Standard curve was performed using bovine serum albumin provided with the same assay kit. Absorbance was measured at 595 nm.

#### Superoxide dismutase, SOD

The enzyme superoxide dismutase was measured following the method by McCord and Fridovich [41]. The reaction mixture was prepared with the following solutions mixed in a 96-well microplate: KPB with EDTA (final concentrations of 50 and 2.5 mM, respectively at pH 7.8), cytochrome c (final concentration of 10 μM; C2506, Sigma), and xanthine (final concentration of 50 μM). This reaction mixture served as the blank, and the uninhibited batch was the reaction mixture plus 20 μL of 0.25 U mL^-1^ xanthine oxidase; the inhibited batch was the reaction mixture with 0.25 U mL^-1^ xanthine oxidase and 40 μL of the gill cell homogenate. The change of absorbance was monitored at 550 nm for 5 min.

Superoxide radicals (produced by the system xanthine/xanthine oxidase) reduce the cytochrome c, and the rate of this reduction is followed spectrophotometrically at 550 nm. The enzyme superoxide dismutase catalyzes the dismutation of superoxide, thus superoxide dismutase inhibits the reduction of cytochrome c by competing for the superoxide radical. This inhibition reaction reflects the activity of the superoxide dismutase, and is calculated as:

$$\frac{\left[\left(\Delta A_{550nm}{\text{min}}^{-1}Uninhibited)-(\Delta A_{550nm}{\text{min}}^{-1}Inhibited\right)\right](100)}{\left(\Delta A_{550nm}{\text{min}}^{-1}Uninhibited)-(\Delta A_{550nm}{\text{min}}^{-1}Blank\right)}=\%inhibition$$

#### Lactate Dehydrogenase, LDH

The assay used for the quantification of this enzyme was reproduced from Zou et al. [38]. LDH-detection assay mix was prepared with 543 μM nitro blue tetrazolium (N5514, Sigma), 814 μM β-NAD^+^ (N7004, Sigma), 20 U mL^-1^ diaphorase (D2197, Sigma) and 50 mM lactic acid sodium salt (BIOLB0571, Astral Scientific) in 10 mM Tris-HCl buffer at pH 8.5. A volume of 50 μL of sample was added to wells of a 96-well microplate, 50 μL of LDH-detection assay mix was also added and incubated for 30 min at room temperature. To stop the reaction, 10 μL of 1 M HCl was added and absorbance measured at 560 nm.

Results of LDH were expressed as percentage of total cellular content (% TCC). Gill cells at the same concentration used for the exposure assays were exposed to GSe medium, detached and ruptured by sonication. Samples were centrifuged at 370 ×g for 5 min. The supernatant was recovered and stored at -20°C until analysis. This treatment represented 100% LDH or Total cellular content.

### Data analysis

Data were analysed for normality and homogeneity of variance with Kolmogorov-Smirnov and Hartley’s F_max_ tests, respectively. One way analysis of variance (ANOVA), followed by post-hoc Newman-Keuls tests, were performed to observe any significant difference among experimental groups. Comparisons among treatments within species were performed for data including gill cell viability; comparisons among treatments across species were performed for data including production of superoxide by algae, superoxide dismutase activity, and lactate dehydrogenase release by gill cells. A significance level of 95% (α = 0.05) was considered for all statistical analysis, which were performed with the software Statistica 12. All figures were created using the software Microsoft Excel 2013; when regression lines were required, they were constructed using the same software. LC_50_ values were calculated according to Alexander et al. [42], and algal biovolume according to Hillebrand et al. [43].

## Results

### Effect of harmful microalgae on gill cell viability

**Fig 1:**
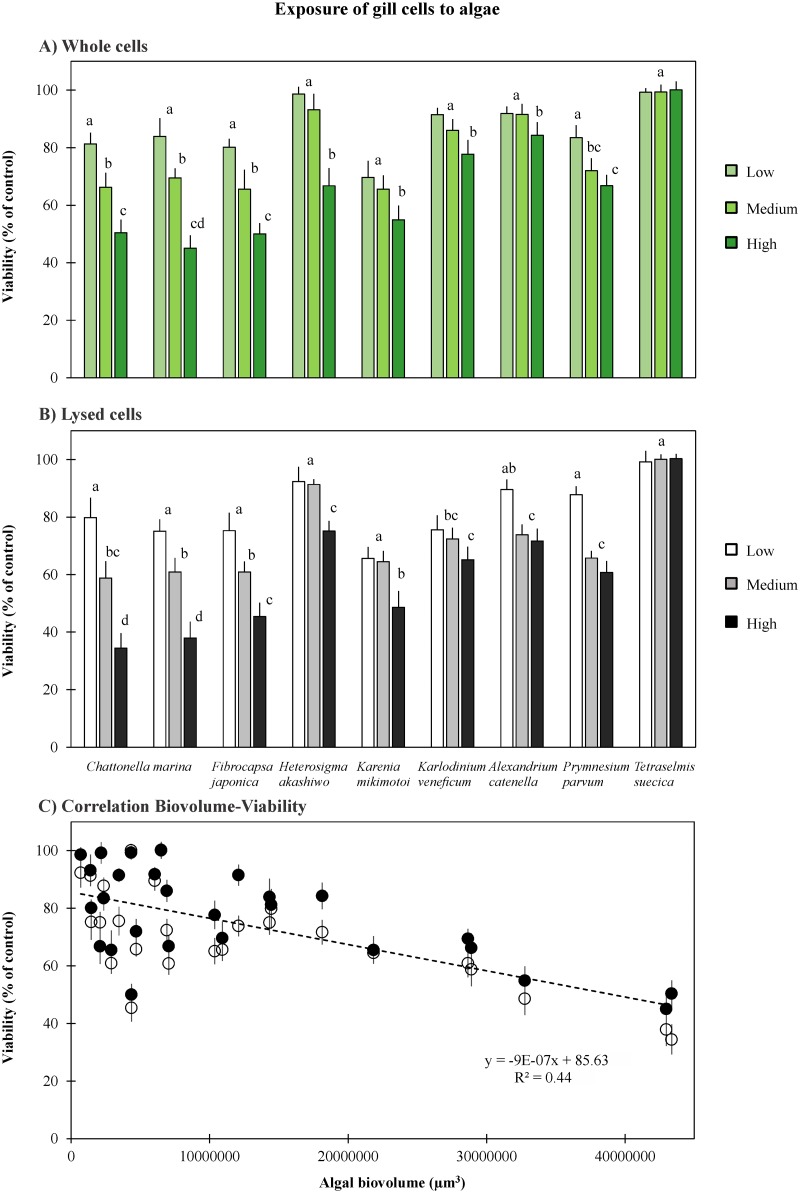
Viability of gill cells RTgill-W1 after a 2-hr exposure to seven species of ichthyotoxic microalgae and one nontoxic. A) Whole algal cells, B) Lysed cells, and C) Correlation between algal biovolume and gill cell viability (blank circles are for lysed algal cells and black circles for whole cells. Trend line and equation correspond to a linear regression). Error bars indicate standard deviations, and letters on top of columns represent significant differences among treatments (whole and lysed cells of three concentrations) within each species. *Chattonella marina* includes the two strains CMPL01 (Australian) and N118 (Japanese). *Tetraselmis suecica* was used as a nontoxic control.

The nontoxic green alga *Tetraselmis suecica* did not exhibit any negative effect on fish gill cell viability (viability or living cells ~100%). All other microalgae exhibited a decrease in gill cell viability after the exposure, showing a higher impact when the algal concentration was increased. In most cases gill cell viability was significantly affected at high algal cell concentrations (up to 65% loss of viability), especially for *Chattonella marina* (both Australian CMPL01 and Japanese N118 strains). Gill cells exposed to lysed CMPL01 showed viability losses of 20% and 65% at low and high algal cell concentrations, respectively, and lysed cells were significantly more toxic than whole algal cells (Fig 1A and 1B) (*p*<0.001). In general, the raphidophytes *C*. *marina*, *Fibrocapsa japonica*, and the dinoflagellate *Karenia mikimotoi* were the most toxic towards gill cells at high algal concentrations, with gill cell viability reduced to 35–49% after the 2-hr exposure. The raphidophyte *Heterosigma akashiwo* and the dinoflagellate *Alexandrium catenella* were the least toxic among the microalgae tested in this study.

Algal concentration was variable among the different species studied, thus algal biovolume was calculated based on algal concentrations. When all data were considered and algal biovolume was plotted against gill cell viability, a strong correlation was not observed (r^2^ = 0.44), suggesting that the decrease in gill cell viability was not a direct stress response due to the difference in high algal biovolume as compared among algal species (Fig 1C). For instance, gill cells were exposed to *Fibrocapsa japonica* and *Chattonella marina* at biovolumes of 0.44×10^7^ and 4.3×10^7^, respectively, and gill cell viability was reduced to 35–45%.

### Superoxide radical production by algae

*Tetraselmis suecica* and *Prymnesium parvum* showed the lowest production of superoxide radicals (≤0.15 pmol cell^-1^ hr^-1^), followed by *Heterosigma akashiwo* and *Karlodinium veneficum* (≤0.49 pmol cell^-1^ hr^-1^). All other species produced more superoxide after cell lysis, particularly at high algal concentrations. Highest O_2_^-^ production by *Fibrocapsa japonica*, *Karenia mikimotoi*, and *Alexandrium catenella* were in the range 1.94–3.29 pmol cell^-1^ hr^-1^, except for lysed cells of *Alexandrium* at high concentration, which produced almost three times more superoxide compared to low lysed-cell concentration (5.63 versus 1.98 pmol cell^-1^ hr^-1^, respectively).

**Fig 2:**
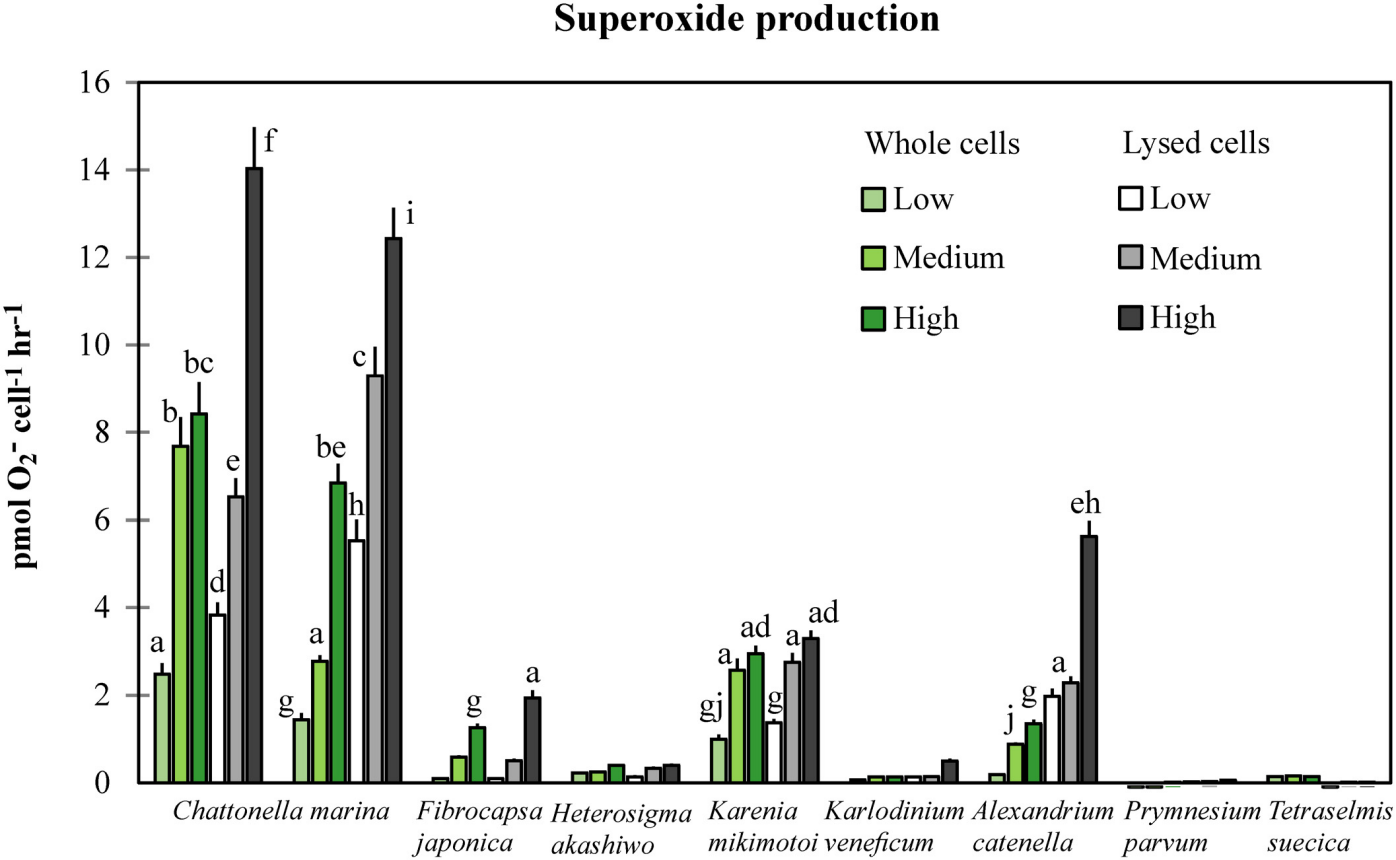
Production of superoxide radicals O2^-^ by algal species under two conditions (whole and lysed cells) at three concentrations each. Error bars represent standard deviations of production rates (n = 3), and letters on top of columns indicate significant differences among all treatments across species with production rates higher than 0.59 pmol cell^-1^ hr^-1^. ANOVA, F_0.05;53,108_ = 441.

*Chattonella marina* was by far the highest producer of superoxide radicals (*p*<0.001), and this increased significantly with increasing algal concentration. Cultures with lysed algal cells showed a higher production of superoxide compared to whole cells, this was 2-fold (N118) and almost 4-fold (CMPL01) higher between low and high algal concentrations. Between the two strains of *C*. *marina* tested, Australian CMPL01 produced significantly more O_2_^-^ than the Japanese N118 upon cell lysis (14.03 and 12.43 pmol cell^-1^ hr^-1^, respectively) (*p* = 0.047) (Fig 2).

### Superoxide dismutase activity in gill cells and its relation to O_2_^-^

**Fig 3:**
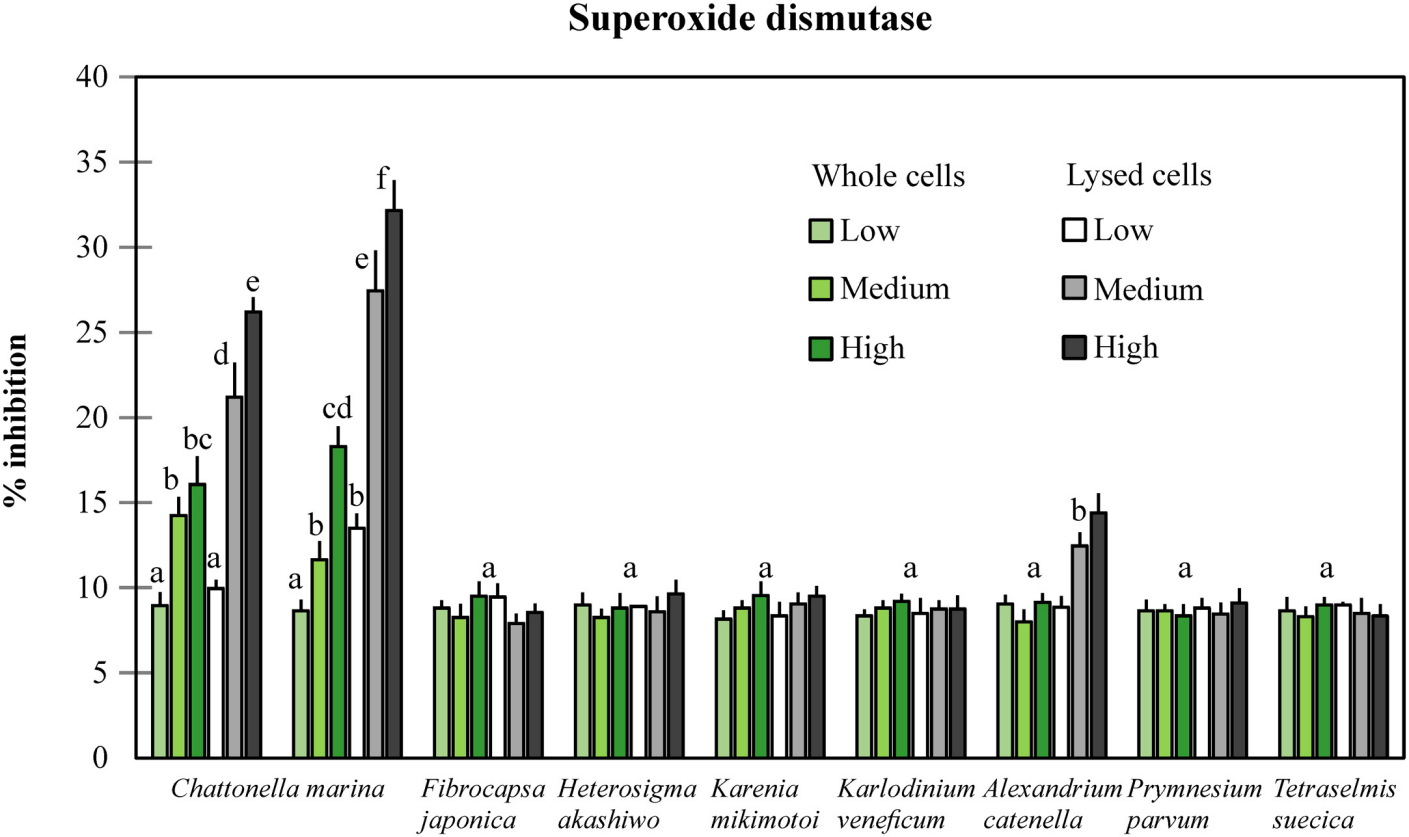
Activity of the enzyme superoxide dismutase from gill cells exposed to toxic algae (except for TSCS187). SOD activity in control cells exposed only to GSe medium was 8.9% inhibition, and activity of gill cells with the letter “a” was not significantly different to control cells. Comparisons were performed among all treatments across species. ANOVA, F_0.05;54,55_ = 70.7.

Activity of superoxide dismutase (SOD) is expressed as the percent inhibition of the reduction of cytochrome c by competing for the superoxide radical. Thus a higher percent inhibition indicates a higher enzyme activity. SOD activity in control gill cells (exposed only to GSe culture medium) was 8.9 (±0.7) % enzyme inhibition. Gill cells did not show significant differences in SOD activity between control and those exposed to the algae *Fibrocapsa japonica*, *Heterosigma akashiwo*, *Karenia mikimotoi*, *Karlodinium veneficum*, *Prymnesium parvum*, *Tetraselmis suecica*, and whole cells of *Alexandrium catenella* (*p*≥0.07). Gill cells showed a significant SOD activity increase upon exposure to medium and high concentrations of lysed cells of *A*. *catenella* (up to 14.4% inhibition; *p*≤0.03). SOD activity was greater after exposure to medium and high concentrations of both strains of *Chattonella marina* CMPL01 and N118 (*p*≤0.04). SOD activity was significantly highest in gill cells exposed to lysed cells of *C*. *marina*, with a maximum activity of 32.2% inhibition (an increase of 23.3% compared to control gill cells; *p* = 0.0002) at high concentrations of the Japanese strain N118 (Fig 3).

**Fig 4:**
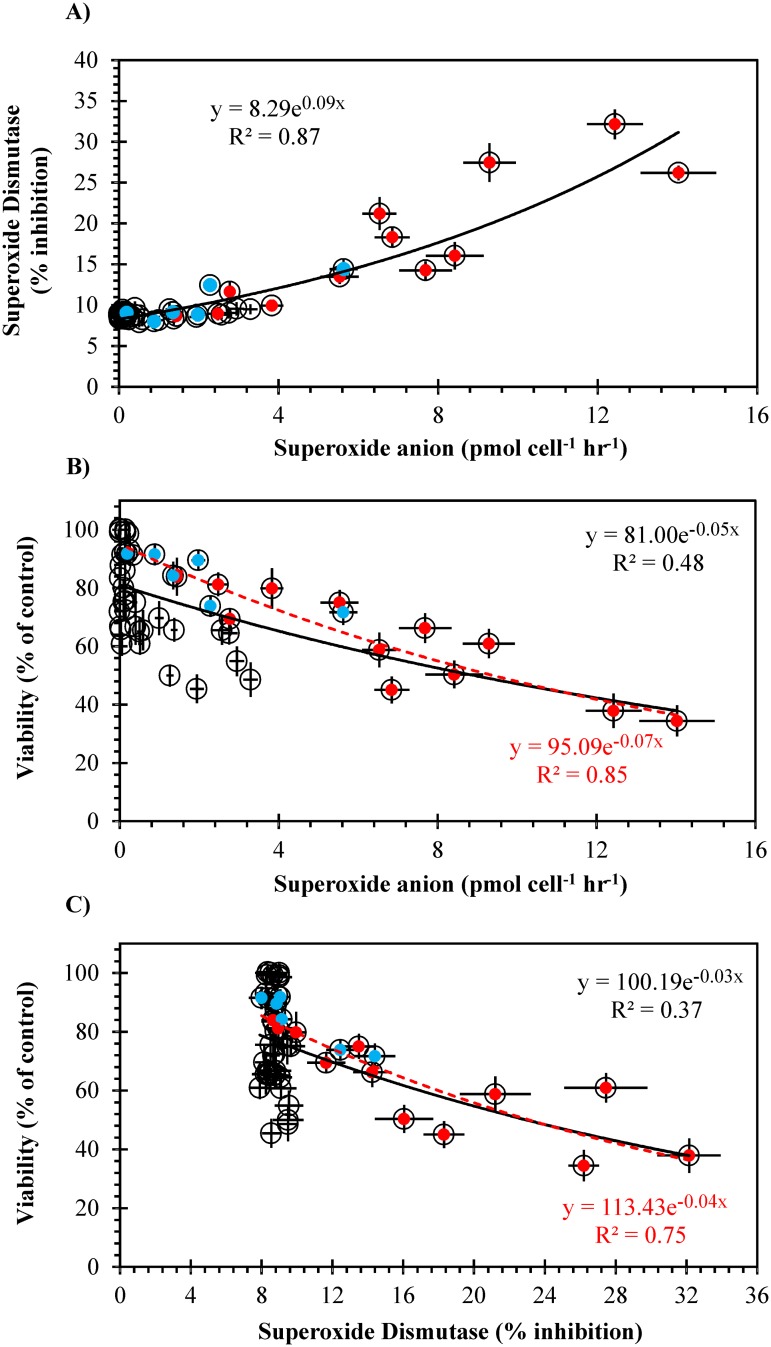
Relationship between (A) superoxide production by algae and SOD activity in gill cells, (B) superoxide production by algae and gill cell viability, and (C) gill cell SOD activity and viability after exposure to algae. Red symbols are for *Chattonella marina*, and blue symbols for *Alexandrium catenella*. Trend lines and equations of the adjustment (exponential regression) are shown for all data (black line and top equation) and data from *C*. *marina* and *A*. *catenella* (red interrupted line and bottom equation in B and C).

A strong relationship existed between superoxide anion production by algae and activity of SOD in gill cells (r^2^ = 0.87). However, the relationship between O_2_^-^ production by algae and gill cell viability, and SOD activity and viability both in gill cells, was not very strong (r^2^≤0.48); only when results from *C*. *marina* and *A*. *catenella* were considered, was this relationship stronger (Fig 4).

### Catalase activity in gill cells

Gill cells did not show any significant changes in catalase (CAT) activity after exposure to algae when compared to controls (exposed only to GSe medium) (data not shown). Control cells had an activity of 0.80 (±0.08) μmol min^-1^ mg protein^-1^; gill cells exposed to the nontoxic alga *Tetraselmis suecica* and all toxic algae showed CAT activities ranging between 0.78 and 0.93 μmol min^-1^ mg protein^-1^.

### Lactate dehydrogenase activity in gill cells

**Fig 5:**
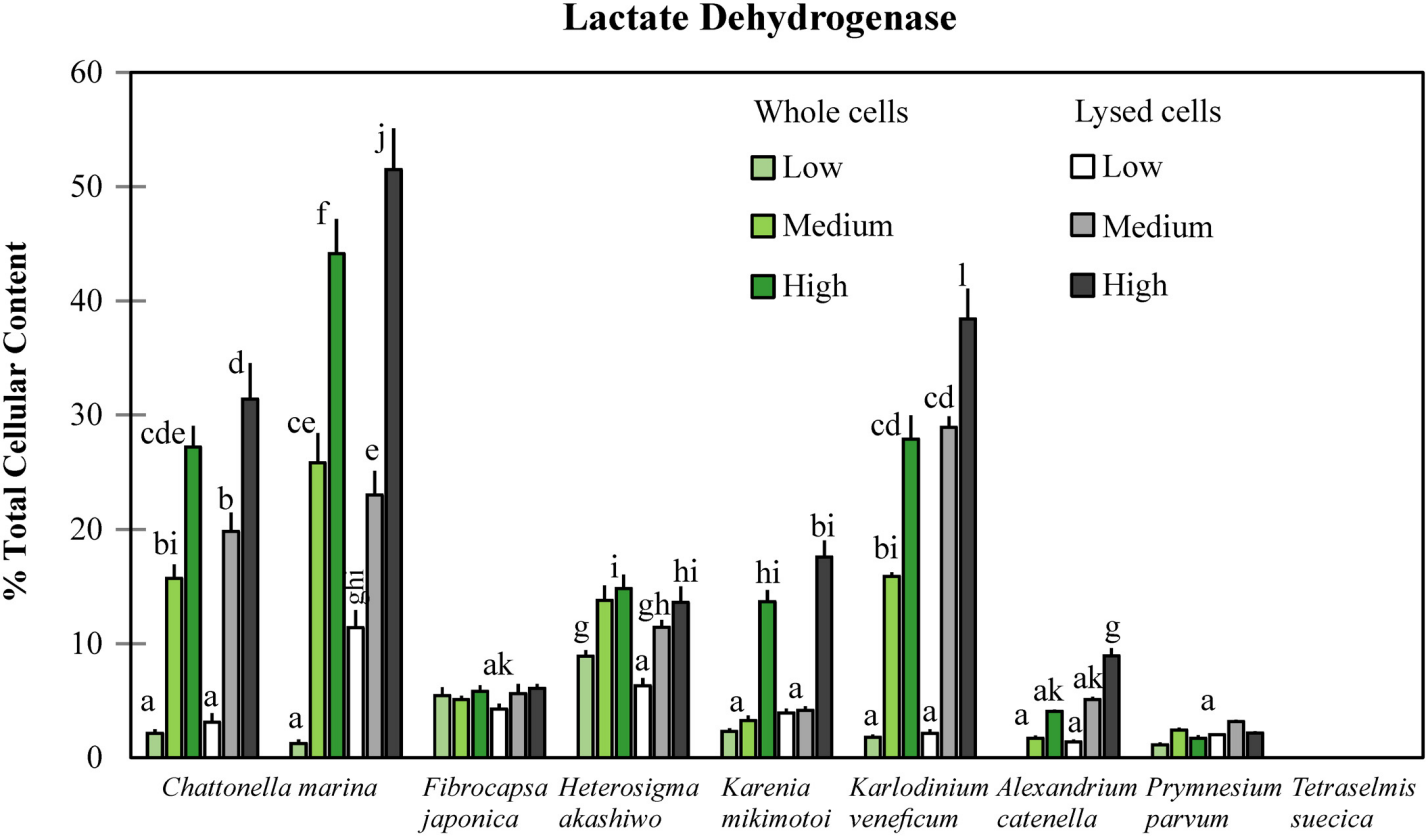
Release of the enzyme lactate dehydrogenase by gill cells (as % of Total Cellular Content) after exposure to harmful microalgae (except TSCS187). Error bars indicate standard deviations and letters on columns show significant differences among all experimental treatments after comparisons were performed across species. ANOVA, F_0.05;54,55_ = 210, *p*<0.001.

**Fig 6:**
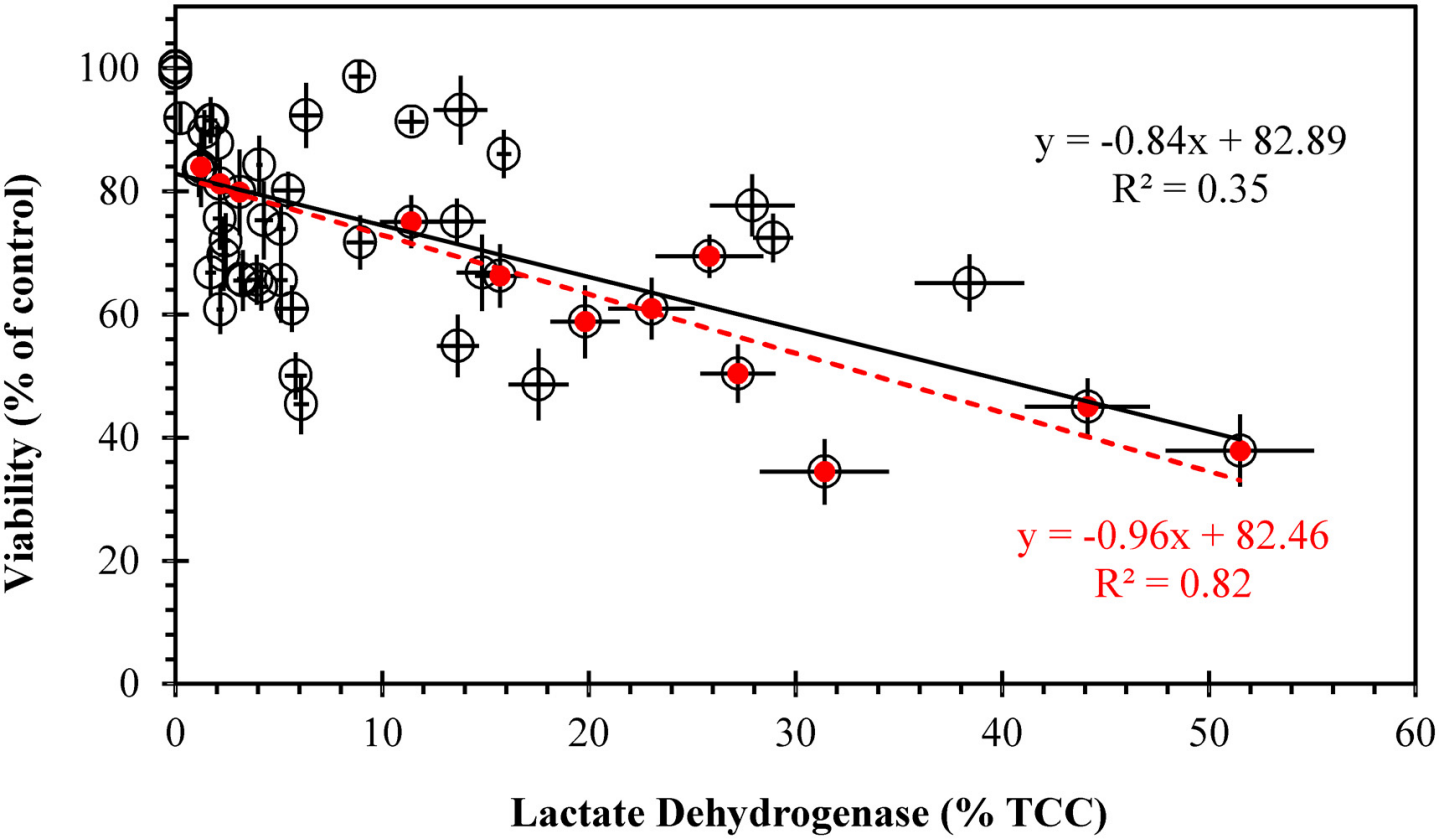
Relationship between LDH-release and viability of gill cells after exposure to microalgae. Red symbols represent *Chattonella marina*. Trend line and equation of linear adjustment are presented for all data (black line and top equation in black) and *C*. *marina* (red interrupted line and bottom equation in red). Error bars indicate standard deviations of LDH (x-axis) and viability (y-axis).

No release of the enzyme lactate dehydrogenase (LDH) was observed on gill cells exposed to *Tetraselmis suecica*. Gill cells exposed to the haptophyte *Prymnesium parvum* showed low and non-significant levels of LDH-release (1.1–3.2% TCC; *p*≥0.86), which also occurred with gill cells exposed to low concentrations of other algal species. More LDH-release was observed when gill cells were exposed to higher algal cell concentrations, especially when these were lysed. Highest LDH-release was detected in gill cells exposed to Japanese *Chattonella marina* N118 (44.1–51.5% TCC; *p*≤0.00025), followed by those exposed to the dinoflagellate *Karlodinium veneficum* (27.9–38.4% TCC; *p*≤0.00020), and Australian *C*. *marina* CMPL01 (27.2–31.4% TCC; *p*≤0.00025) (Fig 5; ANOVA, F_0.05;54,55_ = 210, *p*<0.001). Additionally, there was not a strong relationship observed between gill cell LDH-release and viability (r^2^ = 0.35), except with *C*. *marina* (r^2^ = 0.82) (Fig 6).

### Effect of algal extracts and purified phycotoxins on gill cell viability

**Fig 7:**
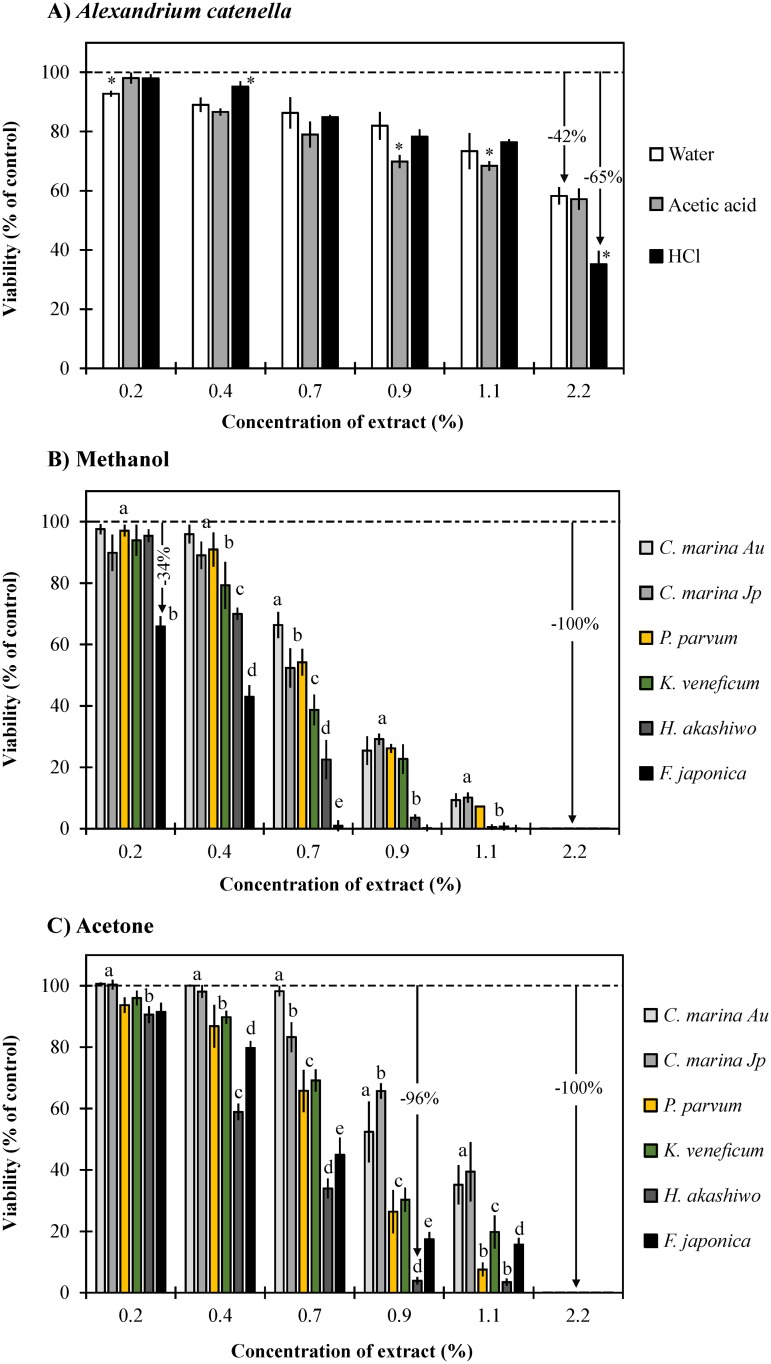
Effect of algal extracts on gill cell viability. Aqueous extracts of *Alexandrium catenella* (A), methanol extracts (B) and acetone extracts (C) of *Chattonella marina*, *Fibrocapsa japonica* and *Heterosigma akashiwo* (raphidophytes), *Prymnesium parvum* (haptophyte), and *Karlodinium veneficum* (dinoflagellate). Error bars represent standard deviations of quadruplicates, and asterisks or letters indicate significant differences among treatments within each concentration tested.

Amongst the eight species tested, algal extracts of *Tetraselmis suecica* (negative control) were not toxic in any of the solvents used. Aqueous extracts of *Prymnesium parvum* were toxic only at the highest concentration (2.2% v/v), decreasing gill cell viability significantly by 22% (*p*<0.0008). Aqueous, acetic acid and hydrochloric acid extracts of *Alexandrium catenella* showed a similar pattern in toxicity, however, the hydrochloric acid extract was more toxic at the highest concentration (42% versus 65% decrease in gill cell viability; *p* = 0.00018; Fig 7a). Methanol and acetone extracts of *A*. *catenella* did not have any effect on gill cell viability; extracts of *Karenia mikimotoi* were only toxic at the highest concentrations, decreasing gill cell viability by 98% (MeOH; *p* = 0.00016) and 71% (acetone; *p* = 0.00016).

Methanol and acetone crude extracts of *Chattonella marina*, *Heterosigma akashiwo*, *Fibrocapsa japonica* (raphidophytes), *Prymnesium parvum* (haptophyte), and *Karlodinium veneficum* (dinoflagellate) decreased gill cell viability with increasing extract concentration. The methanol extract of *F*. *japonica* was significantly the most toxic among these algal species (100% decrease of viability at concentrations >0.7% v/v) (*p* = 0.00016), but *H*. *akashiwo* was the most toxic when acetone was used for the extraction (96% decrease of viability at concentrations ≥0.9%) (*p*≤0.00726). Comparing the efficacy extraction of solvents, methanol extracts were more toxic at lower concentrations, but acetone extracts were equally toxic at the highest concentration (2.2% v/v showed a decrease of viability of 100% in all species; Fig 7B and 7C).

**Fig 8:**
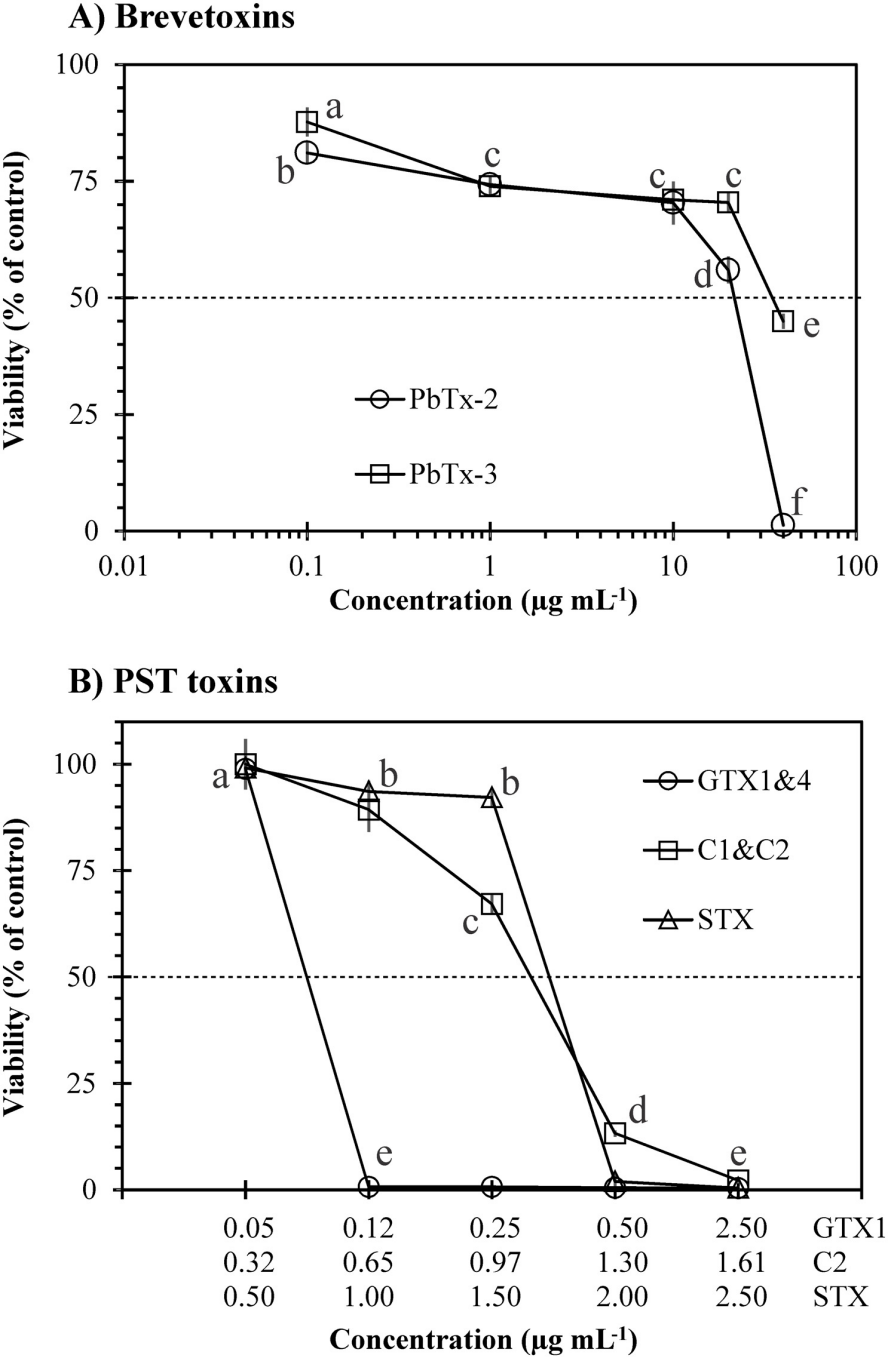
Viability of gill cells after exposure to increasing concentrations of (A) brevetoxins and (B) PST toxins. Error bars represent standard deviations of four replicates. Interrupted lines show LC_50_ values for each phycotoxin: PbTx-2 = 22.1 μg mL^-1^, PbTx-3 = 35.2 μg mL^-1^; GTX1&4 = 0.09 μg mL^-1^ GTX1 with 0.03 μg mL^-1^ GTX4, C1&C2 = 3.58 μg mL^-1^ C1 with 1.07 μg mL^-1^ C2, and STX = 1.71 μg mL^-1^. The epimer of greatest toxicity was included in the x-axis for toxins that are combined (i.e. GTX1 for the mix GTX1&4, and C2 for C1&C2). Letters next to the symbols in each line indicate significant differences among all treatments.

**Fig 9:**
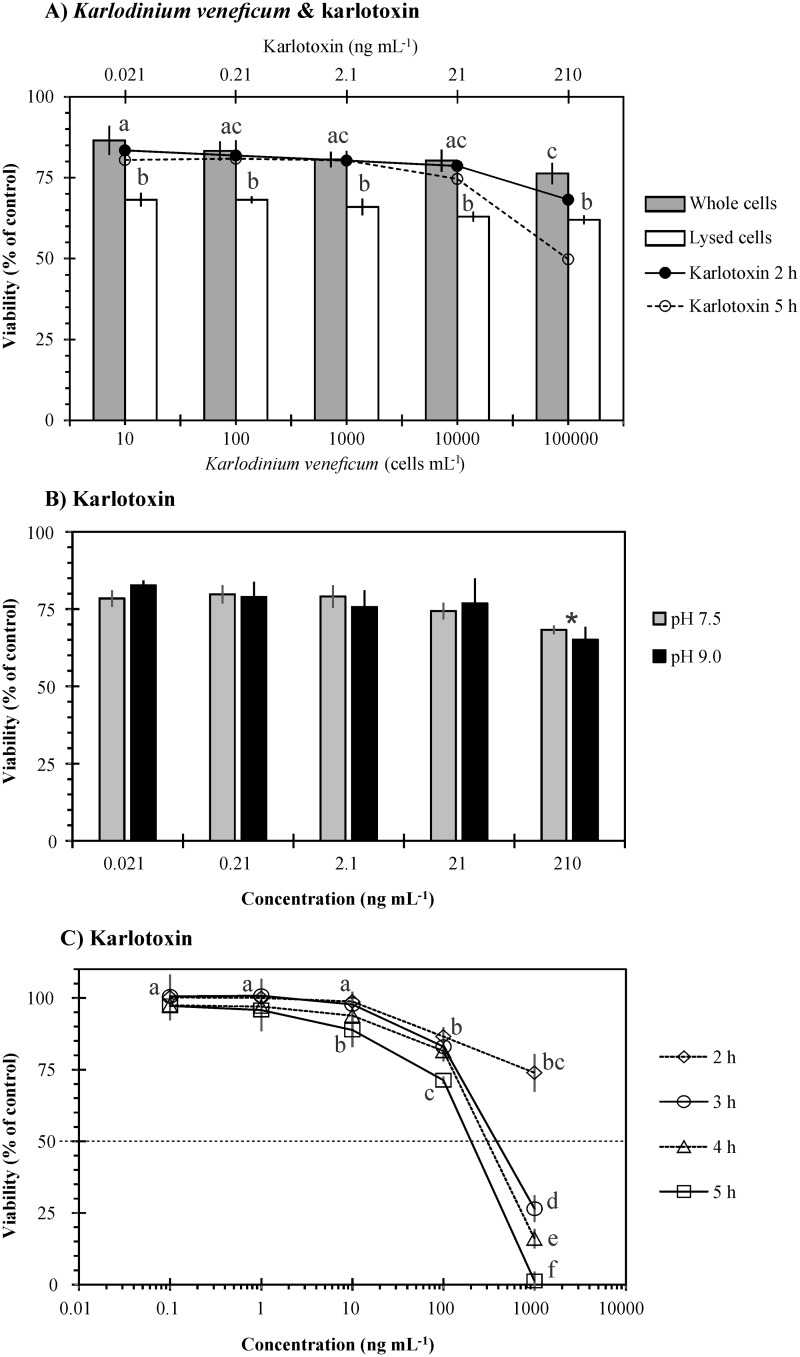
Viability of gill cells after exposure to (A) *Karlodinium veneficum* and equivalent karlotoxin (KmTx-2) concentrations, (B) karlotoxin at different pH, and (C) karlotoxin at higher concentrations. Error bars represent standard deviations of four replicates. The interrupted line in C shows LC_50_ values: 380 (3 hrs), 293 (4 hrs), and 203 ng mL^-1^ (5 hrs). Letters or asterisk on top of columns or next to the symbols in the lines indicate significant differences among treatments.

Purified brevetoxin PbTx-2 was more toxic than PbTx-3, with LC_50_ of 22.1 and 35.2 μg mL^-1^, respectively (Fig 8A). The difference in toxicity was significant at concentrations ≥20 μg mL^-1^ (*p* = 0.0002), PbTx-2 decreased gill cell viability by 44% and PbTx-3 by 30%. A decrease of gill cell viability of 99% and 55% was observed at the highest concentration of PbTx-2 and PbTx-3 (40 μg mL^-1^), respectively (Fig 8A). Among the PST toxins tested, GTX1&4 was the most toxic (*p* = 0.0002), followed by STX and C1&C2. Gonyautoxins 1 and 4 were supplied as a cocktail, as well as C1&2; LC_50_ observed for each toxin were 0.09 μg mL^-1^ GTX1 with 0.03 μg mL^-1^ GTX4, 1.71 μg mL^-1^ for STX, and 3.58 μg mL^-1^ C1 with 1.07 μg mL^-1^ C2 (Fig 8B). Karlotoxin KmTx-2 showed a comparable toxicity with live and lysed *Karlodinium veneficum* cells (<38% loss of viability; Fig 9A). Toxicity of karlotoxin did not vary between pH 7.5 and 9.0 (17–35% loss of viability; Fig 9B). Low toxicity was observed in a 2-hr exposure (≤26% loss of gill cell viability) but a significant increase in toxicity was observed with time, particularly at a concentration of 1000 ng mL^-1^ (Fig 9C) (*p*≤0.0347). Lower LC_50_ values were observed as exposure time was increased. LC_50_ calculated at 3, 4 and 5 hrs were 380, 293, and 203 ng mL^-1^, respectively (previously reported in Place et al. [44]).

A comparison of fatty acid composition (expressed as % of total fatty acids) in the microalgae studied (Table 2) suggests the role of free fatty acid (FFA) and polyunsaturated fatty acids (PUFA), such as eicosapentaenoic (20:5ω3, EPA), octadecatetraenoic (18:4ω3, OTA) and octadecapentaenoic (18:5ω3, OPA) acids, in ichthyotoxicity as has been demonstrated in our previous work [10]. Methanol extracts of *Tetraselmis suecica* did not have any toxicity on gill cells. This alga does not produce high amounts of PUFA and FFA [45], whereas *C*. *marina*, *F*. *japonica*, and *H*. *akashiwo* produce high amounts of OTA and EPA. The methanol extract of *F*. *japonica* was the most toxic (≥99% decrease of gill cell viability at 0.7% of extract concentration) (*p* = 0.00016), and this species appears to produce the highest amount of FFA (38% of total lipids; [46] Table 2).

**Table 2:** Fatty acid composition (as % of total fatty acids) in red tide phytoplankton.

	*Tetraselmis suecica* ^a^	*Chattonella marina* Au^b^	*C*. *marina* Jp^b^	*Heterosigma akashiwo* ^c^	*Fibrocapsa japonica* ^c^	*Prymnesium parvum* ^d^	*Karlodinium veneficum* ^e^	*Karenia mikimotoi* ^e^
**Saturates**								
14:0	0.9	8.1	6.1	5.8	18.5		14.0	14.1
16:0, PA	24.0	21.3	20.7	22.8	7.6	37.6	16.0	23.8
18:0	0.6	0.7	0.6	0.6	1.6	8.6	2.6	2.5
**Monounsaturates**								
16:1ω7c	0.3	7.1	7.1	7.2	2.6	2.6	3.7	3.6
16:1ω13trans	0.8	2.9	5.1	3.3	1.2			
18:1ω9c	14.5	2.5	2.3	5.0	7.4	21.7	6.7	5.2
18:1ω7c	1.1	0.6	0.5	1.0	0.5		1.8	1.2
**Polyunsaturates**								
18:4ω3 OTA	4.8	**18.4**	**20.5**	**19.5**	**26.6**	4.5	2.3	0.5
18:2ω6 LA	13.9	2.5	3.1	1.7	2.9	8.7	0.5	3.3
18:3ω3 ALA	4.6	3.5	3.3			1.8		
18:5ω3 OPA	-			6.7	0.0		**34.9**	**21.3**
20:4ω6 ARA	2.1	1.9	3.7	0.8	4.4			
**20:5ω3 EPA**	5.3	**23.0**	**21.5**	**18.7**	**17.4**	0.5	0.9	1.1
22:5ω6 DPA		2.4	1.5	0.1	0.6		0.1	0.1
22:6ω3 DHA	<0.2	2.7	1.2	3.1	0.4	8.9	10.3	10.2
Sum SFA	26.8	30.1	27.4	29.2	27.6		33.8	42.1
Sum MUFA	20.5	13.1	15.0	16.5	11.6		14.8	13.2
Sum PUFA	49.7	54.4	54.8	51.7	54.5		51.4	44.6
Free Fatty Acids*	0.8	4.6	3.7	2.0	**37.9**		8.6	3.3

^a^ Data from Volkman et al. [45];

^b^ Dorantes-Aranda et al. [27];

^c^ Marshall et al. [46];

^d^ Makri et al. [47];

^e^ Mooney et al. [48].

* Free fatty acid composition as % of total lipids.

Fatty acid abbreviations: palmitic acid, PA; octadecatetraenoic acid, OTA; linoleic acid, LA; α-linolenic acid, ALA; octadecapentaenoic acid, OPA; arachidonic acids, ARA; eicosapentaenoic acid, EPA; docosapentaenoic acid, DPA; docosahexaenoic acid, DHA; saturated fatty acids, SFA; monounsaturated fatty acids, MUFA; polyunsaturated fatty acids, PUFA.

## Discussion

In the present work, application of an *in vitro* fish gill assay has allowed us to carry out sensitive screening tests for toxicity of living and lysed marine microalgae and their extracts. Combined with the simultaneous assessment of SOD, CAT and LDH enzyme activities on fish gill cells, this offered the opportunity to start partitioning the role of reactive oxygen radicals, lipid fractions and phycotoxins in causing ichthyotoxicity.

We previously reported high ichthyotoxicity of *Chattonella marina* (CMPL01 and N118) and *Karlodinium veneficum* (KVSR01) towards RTgill-W1 cells [10,27,30]. In the present study five further microalgae were added, including the raphidophytes *Heterosigma akashiwo* and *Fibrocapsa japonica*, the dinoflagellates *Karenia mikimotoi* and *Alexandrium catenella*, the haptophyte *Prymnesium parvum*, and we also used the green alga *Tetraselmis suecica* as a nontoxic negative control. We confirmed that ichthyotoxic microalgae are generally more toxic after cell lysis. Gill cells showed lower viability upon exposure to lysed algal cells, except for the raphidophyte *Heterosigma akashiwo* (HAGB01), which was more toxic when cells were intact possibly due to a highly labile chemical compound [49]. The armoured dinoflagellate *Karlodinium* is essentially nontoxic, except under conditions that cause cell lysis [26]. *C*. *marina* was the most ichthyotoxic species, with no significant differences between Australian (CMPL01) and Japanese (N118) strains.

*Chattonella marina* was the greatest producer of the radical superoxide, which confirms previous findings by Oda et al. [11] and Marshall et al. [21] that at least for this species ROS are a key driver of ichthyotoxicity. However, comparing a broader range of microalgae showed there was not a strong relationship between O_2_^-^ generation by algae and gill cell viability (Fig 4B). Critically, our previous work showed that O_2_^-^ produced artificially via the enzymatic system xanthine/xanthine oxidase (equivalent to *C*. *marina* levels), only affected gill cell viability by 14% [10]. This finding strongly suggests that ROS do not damage fish gill cells in their own right, but rather through synergistic reactions with other compounds, such as lipids through lipid peroxidation [12]. Lipid peroxidation is a set of chain reactions, especially for polyunsaturated fatty acids because of their double bonds, where hydrogen atoms are abstracted from the lipid molecules. Since a hydrogen atom has only one electron, this leaves behind an unpaired electron on the carbon atom. This carbon radical is usually stabilized by a molecular rearrangement to produce a conjugated diene, which rapidly reacts with O_2_ to produce a hydroperoxy radical, which abstracts hydrogen atoms from other lipid molecules and the chain reaction of lipid peroxidation continues [50,51]. In raphidophytes, ROS interactions with EPA can account for most of the ichthyotoxicity [12], while our recent observations show that for *Alexandrium catenella*, ROS interactions with DHA play a key role. Among the products of lipid oxidation reactions that show adverse effects *in vivo* and *in vitro* are aldehydes, lipid radicals and lipid hydroperoxides [52,53]. These compounds are highly reactive and able to attack cell membranes and affect cell functions that may lead to death [28,51]. Our previous study showed toxicity by some aldehydes towards gill cells [27]; although the aldehydes tested were commercial preparations, aldehydes have been found in diatoms and haptophytes as a chemical defense against grazers [54,55].

The two enzymatic biomarkers of oxidative stress, catalase and superoxide dismutase, were assessed directly in the gill cells after exposure to the microalgae. Catalase catalyses the decomposition of hydrogen peroxide (H_2_O_2_) to water and oxygen, and thus its activity is directly associated with the effect of hydrogen peroxide in oxidative stress. Oda et al. [11] found high levels of H_2_O_2_ in *C*. *marina*; however, our results showed no changes in catalase activity between control and experimental cells (CAT levels were maintained in the range 0.78–0.93 μmol min^-1^ mg protein^-1^). Tang et al. [56] observed that the effects of fish exposed to *C*. *marina* and H_2_O_2_ itself were not comparable, concluding that H_2_O_2_ was not the cause of fish mortality. This was also observed by Twiner et al. [57], who found that levels of H_2_O_2_ produced by *Heterosigma akashiwo* are not able to kill the brine shrimp *Artemia salina* or vertebrate cells. It remains possible that H_2_O_2_ produced by *C*. *marina* quickly reacts with metals such as iron to produce other ROS via the Fenton reaction. The reduced form of iron (Fe^2+^) reacts with H_2_O_2_ and produces hydroxyl radicals, which are highly reactive and can also cause oxidative stress in organisms; the oxidized form of iron (Fe^3+^) also reacts with H_2_O_2_ and produces superoxide radicals [50]. These findings confirm that H_2_O_2_ on its own is not ichthyotoxic, but can produce more toxic ROS in the presence of iron, which commonly occurs in high concentrations in coastal waters [58] and as was present in our GSe medium (~5.4 μM). This may also explain the higher production of superoxide by lysed algal cells compared to intact cells, as Oda et al. [11] found that lysed cells produced more H_2_O_2_ than intact cells, which may quickly react with iron to produce superoxide radicals. Superoxide dismutase catalyzes the dismutation of superoxide anion into oxygen and H_2_O_2_, and thus the effect of superoxide is reflected in SOD activity. SOD in gill cells was induced after exposure to high concentrations of *Alexandrium catenella* (14.4% inhibition) and particularly to *C*. *marina* (32.2% inhibition), but it remained the same as the control (nontoxic *Tetraselmis suecica*) and for samples exposed to other harmful microalgae. There was a good relationship between O_2_^-^ production by algae and SOD activity in gill cells (Fig 4A), indicating that SOD is a good biomarker for this radical. However, the relationship between O_2_^-^ and gill cell viability (Fig 4B), and SOD activity and gill cell viability (Fig 4C) was not significant, especially because when O_2_^-^ production was low or nil, and SOD activity was normal, gill cell viability was variable (40–100%). However, this relationship was considerably stronger when only results from *C*. *marina* and *A*. *catenella* were considered. Superoxide radicals are not the only ROS, but they have become the most practical to measure ROS species due to the difficulty to quantify the most potent radical, hydroxyl, in algal cultures.

Our findings support in part the conclusion by Woo et al. [59], who found that ROS are not the main toxic principle by *C*. *marina*, since oxidative stress was not observed on gills and erythrocytes of red sea bream (*Rhabdosarga sarba*) after exposure to *C*. *marina*, but it did occur when ROS (only as H_2_O_2_) were used for the exposure experiment. It might be the case that our *in vitro* assay is more sensitive than the *in vivo* assay Woo et al. performed, or that algae were used under normal culture conditions and not under other circumstances when they produce more superoxide and free fatty acids, such as when cells are lysed [27]. Also, these authors only used H_2_O_2_ as a ROS species, and in our work we did not find CAT activity changes that could indicate the involvement of H_2_O_2_.

The observed effects of ichthyotoxic microalgae on gill cells in this study are summarized in Table 3. We conclude that both strains of *C*. *marina* produced the highest levels of superoxide, caused the lowest viability on gill cells, and concurrently enzymatic activity of superoxide dismutase and lactate dehydrogenase in gill cells significantly increased. Similar effects were observed on gill cells after exposure to the dinoflagellate *A*. *catenella*, although the impacts were lower. It is clear that ichthyotoxic species do not always share common mechanisms. For instance, it is now well established that the dinoflagellate *Karlodinium veneficum* produces karlotoxins that affect gill cell viability [44]. The methanol extract of *K*. *veneficum* was the most toxic towards gill cells, and this result can quantitatively be attributed to the ichthyotoxicity of purified karlotoxin produced by this strain [60] since methanol is used to extract this toxin [61]. *A*. *catenella* produces a cocktail of hydrophilic PST toxins (neosaxitoxin and gonyautoxins [62]), but purified PST does not quantitatively account for ichthyotoxicity using our *in vitro* model since our recent experiments show that toxin concentrations equivalent to those produced by this alga affected gill cell viability by less than 30%, and the toxin concentrations used in the present study exceed the production levels observed in *A*. *catenella*. However, these observations still need to be confirmed with *in vivo* experiments. Superoxide radicals contributed only a small fraction (~6%) since a slight increase in production of superoxide and SOD activity was observed on gill cells. However, we observed that only aqueous, acetic acid and HCl extracts of *A*. *catenella* were toxic to gill cells (causing up to 65% decrease of gill cell viability), suggesting an important role of highly hydrophilic compounds. However, other lytic compounds are suspected to be the major culprit of *Alexandrium* (i.e. *A*. *tamarense*) mediated fish kills [63,64]. Although their chemical nature and structure have not been fully described yet, it has been found that they are amphipathic compounds (with polar and non-polar components) extracted only with a mix of n-hexane and water [64].

**Table 3:** Summary of impacts of microalgae on fish gill cells after a 2-hr exposure. Highest values are in bold.

Algae species	Strain	Highest concentration (cells mL^-1^)*	Superoxide production (pmol cell^-1^ hr^-1^)	Gill cell viability (% of control)**	Superoxide dismutase (% inhibition)	Lactate dehydrogenase (% TCC)
*Tetraselmis suecica*	TSCS187	147,500	0.0–0.15	100	8.3–9.0	0
*Chattonella marina*	CMPL01	28,750	2.48–**14.03**	**35**	9.0–**26.2**	2.2–**31.4**
	N118	28,500	1.43–**12.43**	**38**	8.7–**32.2**	1.2–**51.5**
*Fibrocapsa japonica*	FJCS332	16,250	0.01–1.94	**46**	8.3–9.5	4.3–6.1
*Heterosigma akashiwo*	HAGB01	40,000	0.13–0.40	67***	8.3–9.7	6.3–14.8
*Karenia mikimotoi*	KMWL01	26,250	0.99–3.29	49	8.2–9.6	2.3–17.6
*Karlodinium veneficum*	KVSR01	130,000	0.06–0.49	65	8.4–9.2	1.8–**38.4**
*Alexandrium catenella*	ACCH05	30,000	0.19–**5.63**	72	8.0–**14.4**	0.2–8.9
*Prymnesium parvum*	PPDW02	572,500	0.0–0.06	61	8.4–9.1	1.1–3.2

* Highest concentration of cells in exponential growth phase; medium and low cell concentrations were 2/3 and 1/3, respectively.

** Gill cell viability corresponds to the lowest values relative to controls observed after exposure.

*** Only strain in which whole algal cells were more toxic than ruptured cells.

LDH-release by gill cells proved to be a good indicator of RTgill-W1 cell viability only for *C*. *marina*, which showed the highest LDH-release, reflecting membrane damage that confirms our previous findings where micrographs of gill cells taken by scanning electron microscopy showed membrane disruption after exposure to *C*. *marina* [30]. Gill cells exposed to the nontoxic alga *Tetraselmis suecica* did not exhibit any LDH-release, but exposure to other toxic algae did. Thus, membrane damage may not be the direct cause of viability changes but it may trigger other physiological changes that alter gill cell viability, such as ion exchange alteration, causing an increase in membrane permeability that may result in cell death through osmotic shock. This mechanism has been suggested for the dinoflagellate *Karlodinium veneficum* (LDH release of 38.4% TCC), whose karlotoxins cause pore-forming disruptions in membranes containing cholesterol that ultimately lead to cellular hypertrophy and osmotic cell lysis, which has been observed on epithelial and chloride cells of fish gills [13].

The hypothesis of synergy between superoxide radicals and other ichthyotoxic compounds was first proposed by Marshall et al. [12], showing that free fatty acids occurring together with superoxide accelerated fish mortality threefold. Our previous work showed that *C*. *marina* was able to produce high levels of free fatty acids (up to 33.2% of total lipids) and superoxide under conditions when rapid changes occurred, such as cell lysis and changes from dark to light conditions (simulating vertical migration) [27]. Aqueous extracts of the raphidophytes *Chattonella marina*, *Fibrocapsa japonica*, and *Heterosigma akashiwo* did not show any effects on gill cell viability, but comparable methanol and acetone extracts were highly toxic, suggesting the role of hydrophobic compounds, such as fatty acids, in their toxic mechanism. Methanol is one of the solvents used for lipid extraction [65], and despite being a polar solvent, it is able to extract FFA to some extent. The Bligh and Dyer method is the preferred protocol for lipid extraction worldwide due to its simplicity and efficiency. It consists of a solvent mix of chloroform/methanol/water where the samples are left overnight, and phases separated by adding more chloroform/water. Most of the lipids are recovered from the chloroform (lower) layer, but a study employing different solvent combinations of this standard method, showed that increasing the methanol proportion in the solvent mix, also increased lipid extraction [66];, however another study found that when increasing the methanol proportion, the fatty acid extraction efficiency remained the same [67]. Hemolytic compounds have been purified from methanol extracts of *F*. *japonica*, and identified as PUFA [68,69]. Additionally, a fraction in 100% methanol purified from *Prymnesium parvum* was found to retain all the ichthyotoxic activity [9]. It has been demonstrated that pH affects toxicity of fatty acid amides and fatty acids [70]. While early work reported that *Prymnesium* ichthyotoxicity only occurred at pH ≥7.0 [71], the extent of toxicity did not increase between pH 7.5 and 9.0 [72]. However, Ulitzur and Shilo [73] reported the opposite trend (increased toxicity at higher pH). Our recent observations during the exposure of gill cells to lysed *Prymnesium* at pH 7 and 9 did not detect any differences in toxicity. Interestingly, in the present study, we did not observe any effect of pH (7.5 versus 9.0) on karlotoxin potency, showing that the role of pH in ichthyotoxicity is not universal.

Table 4 summarises the effects of live algae, algal extracts, toxins and other proposed toxic compounds that have been tested so far on the fish gill cell line RTgill-W1. Superoxide affected gill cell viability by only 14%; in contrast, purified karlotoxin KmTx-2 and brevetoxin PbTx-2 decreased gill cell viability by 99%. Fractions of free fatty acids extracted from *Chattonella marina* also decreased gill cell viability by 100%, as well as methanol and acetone crude extracts of the raphidophytes *C*. *marina*, *Heterosigma akashiwo*, *Fibrocapsa japonica*, and the haptophyte *Prymnesium parvum*.

**Table 4:** Summary of impacts of algae or toxins and substances produced by ichthyotoxic phytoplankton on viability of gill cells (RTgill-W1).

Alga, toxin or chemical	Source	Concentration	Decrease in gill cell viability (%)	Reference
*Chattonella marina*	Live culture, strain CMPL01	7×10^6^ cells L^-1^	55–71%	Dorantes-Aranda et al. [30]
Superoxide radicals (O_2_^-^)	Xanthine/Xanthine oxidase	5–25 μM Xanthine + 30 U L^-1^ Xanthine oxidase	≤14%	Mooney et al. [10]
Eicosapentaenoic acid (EPA)	Commercial preparation (METRAYA)	0.02–120 mg L^-1^	44–98.5%	Mooney et al. [10]
Free fatty acid fractions	Extracted from *Chattonella marina*	0.44 μg mL^-1^	50%	Dorantes-Aranda et al. [27]
		>3.17 μg mL^-1^	100%	
*Karlodinium veneficum*	Live culture, strain KVSR01	1×10^8^ cells L^-1^	24–38%	Dorantes-Aranda et al. [30]
OPA-rich MGDG^+^	Extracted from *Karlodinium veneficum*	0.02–120 mg L^-1^	0–45%	Mooney et al. [10]
OTA-rich MGDG^++^	Extracted from *Amphidinium carterae*	0.02–120 mg L^-1^	17–37%	Mooney et al. [10]
Karlotoxin (KmTx-2)^	Purified from *Karlodinium veneficum*	0.1–1000 ng mL^-1^	3–99%	Place et al. [44] and present study (Fig 9)
2*E*,4*E*-decadienal	Commercial preparation (SIGMA)	0.34 μg mL^-1^	50%	Dorantes-Aranda et al. [27]
2*E*,4*E*-heptadienal	Commercial preparation (SIGMA)	0.36 μg mL^-1^	50%	Dorantes-Aranda et al. [27]
*Alexandrium catenella* extracts	Strain ACCH05 extracted in:			Present study (Fig 7A)
	Water	0.2–2.2% v/v	7–42%	
	Acetic acid 30 mM	=	2–43%	
	Hydrochloric acid 1 mM	=	2–65%	
PST toxins	National Research Council Canada			Present study (Fig 8B)
GTX1&4	Purified from *Alexandrium minutum* Strain AL1V	0.05–2.48 μg mL^-1^ GTX1 0.02–0.81 μg mL^-1^ GTX4	1–99.6%	
C1&C2	Purified from *Alexandrium tamarense* Strain AL18b	1.08–5.40 μg mL^-1^ C1 0.32–1.61 μg mL^-1^ C2	0–97.9%	
STX	=	0.50–2.47 μg mL^-1^	0.9–99.6%	
Methanol extracts:	Extracted in MeOH 99%			Present study (Fig 7B)
*Chattonella marina*	Au CMPL01	0.2–2.2% v/v	2–100%	
	Jp N-118	=	10–100%	
*Prymnesium parvum*	PPDW02	=	3–100%	
*Karlodinium veneficum*	KVSR01	=	6–100%	
*Heterosigma akashiwo*	HAGB01	=	5–100%	
*Fibrocapsa japonica*	FJCS332	=	34–100%	
Acetone extracts:	Extracted in Acetone 80%			Present study (Fig 7C)
*Chattonella marina*	Au CMPL01	0.2–2.2% v/v	0–100%	
	Jp N-118	=	0–100%	
*Prymnesium parvum*	PPDW02	=	6–100%	
*Karlodinium veneficum*	KVSR01	=	4–100%	
*Heterosigma akashiwo*	HAGB01	=	9–100%	
*Fibrocapsa japonica*	FJCS332	=	9–100%	
Brevetoxin (PbTx-2)	Purified from *Karenia brevis* (MARBIONC, USA)	0.1–40 μg mL^-1^	19–99%	Present study (Fig 8A)
Brevetoxin (PbTx-3)	Semisynthetic derivate from *K*. *brevis* (MARBIONC, USA)	0.1–40 μg mL^-1^	12–55%	Present study (Fig 8A)

* Equivalent to production rates by *Chattonella marina* at 6×10^6^ cells L^-1^.

**Containing myristic (14:0, 11%), palmitic (16:0, 25%), palmitoleic (16:1ω7c, 11%), octadecatetraenoic (18:4ω3, 16%), and eicosapentaenoic (20:5ω3, 16%) acids.

^+^Octadecapentaenoic acid (Monogalactosyl diglyceride) 73%, with palmitic acid 26%

^++^Octadecatetraenoic acid 49%, with eicosapentaenoic acid 49%, and docosahexaenoic acid 2%

^^^Provided by Allen R. Place

In conclusion, our results contribute towards increasing the overall understanding of ichthyotoxicity of the microalgal species included in this study. Reactive oxygen species play an important role only with *Chattonella marina*; these ROS may also cause a synergistic effect with the lipids in the alga, producing other toxic compounds through lipid peroxidation (i.e. aldehydes and lipid radicals), which can cause physiological changes in gill cells by increasing superoxide dismutase activity, and damaging gill cell membranes. Karlotoxin accounts for toxicity by *Karlodinium veneficum*, and brevetoxins for *Karenia brevis* (but not *K*. *mikimotoi*); however, PST toxins are not the only toxic component in *Alexandrium catenella*. Other unknown compounds are involved in ichthyotoxicity by *Prymnesium parvum*, *Heterosigma akashiwo*, *Fibrocapsa japonica*, *Alexandrium catenella* and *Chattonella marina*, some of which clearly have a lipid component. Algal cell lysis of fragile fish killing HAB species is critical for ichthyotoxicity to occur, and environmental and physiological conditions (e.g. osmotic shock, turbulence, algal senescence, nutrient deficiency) that enhance this process deserve more attention in defining high alert situations for finfish aquaculture monitoring programmes.
